# Head Movement Synchrony and Idea Generation Interference – Investigating Background Music Effects on Group Creativity

**DOI:** 10.3389/fpsyg.2019.02577

**Published:** 2019-11-15

**Authors:** Sarinasadat Hosseini, Xiaoqi Deng, Yoshihiro Miyake, Takayuki Nozawa

**Affiliations:** ^1^Department of Computer Science, Tokyo Institute of Technology, Kanagawa, Japan; ^2^Research Institute for the Earth Inclusive Sensing, Tokyo Institute of Technology, Tokyo, Japan

**Keywords:** background music, communication, group, creativity, head movement synchrony, idea generation, idea sharing

## Abstract

Previous studies have indicated that divergent idea integration is an effective way to foster extraordinary creativity in groups. This study posits that background music (BGM) may aid in eliciting this phenomenon. Here to describe the effectiveness of BGM on group creativity, we hypothesized and suggested different mechanisms that genre and valence attributes of BGM would lead to extraordinary creativity. The temporal co-ordination of head movement synchrony (HMS) was investigated as a non-verbal cue and we found significant HMS response levels to idea generation. While the HMS as response did not depend on the quality of the prior ideas; it led to higher divergence and originality in the successively generated ideas. Results of this study showed the dominant contribution of upbeat positive valence (UP) music, relative to other genres, in HMS leading to divergent ideas. Following this, the potential role of upbeat music in enhancing participant sociability and positive valence in enhancing cooperation level was discussed. Upbeat positive music may decrease judgmental behavior during creative group tasks and inspire participants to share divergent perspectives. The use of such music can encourage participants to share new perspectives and integrate ideas. It may also provide a potential explanation for the enhancing effect of upbeat positive music on creative outcomes in groups.

## Introduction

Creativity is the development of influential original ideas (Paulus and Nijstad, 2003). Group creativity can be considered a clear illustration of goal-oriented social interaction. Group idea generation and brainstorm sessions are commonly used tools to foster the creative process in educational and industrial settings (Elias et al., 2011). Although brainstorming and group creativity demonstratively facilitate creative outcomes; barriers such as social anxiety and social loafing arguably reduce the efficiency of such processes (McCauley, 1998). The hindered performance can be interpreted by the motivation aspect during teamwork. Group creativity is a group task, and group task outcomes will be based on the output of two major factors – (i) the motivation of individuals to cooperate to perform the task (Slavin, 1983) and (ii) the capacity of individual group members to complete the task. The motivation to co-operate is necessary for a group to function as a system that is more than a mere collection of people placed together, and can be viewed as constituting a system of social interactions (Hare et al., 1955).

Here in this study, we introduced background music (BGM) as a factor to foster group creativity. Despite many studies have been conducted on the effects of music, as music comprises an important part of our daily life, most studies have focused on individual aspects (Rauscher et al., 1995; Thompson et al., 2001; Waterhouse, 2006). Although the impact of music on individual creativity is still under investigation and not based on a unified perspective (Schellenberg, 2015), one recent article highlighted how positive valence classical pieces enhance divergent creativity (Ritter and Ferguson, 2017). On the other hand, studies support the potential effect of music on social interaction – and thus goal-orientated group achievements – including the impact of music on task involvement and the capacity to cope with perceived stress (Fried and Berkowitz, 1979),which would affect communication (Schabracq and Cooper, 2000), and reinforcing social interaction (Cross and Morley, 2009). Consequently, while the potential impact of music on group coordination, and thus group creativity, in varying ways have been indicated, there has been scarce research examining the mechanisms that may underlie music’s impact on group creativity outcomes. Building on our previous work on music (Hosseini et al., 2019), this study firstly confirms the effects of music on group creativity indices, with a larger sample. More importantly, this study explores the temporally refined process that underlies the observed effects of music on group creativity, by positing dynamic non-verbal behavior as a possible determinant of creative interaction. In the following further explanation on non-verbal behavior and its possible connection to group creativity is provided.

As social animals, humans share and receive static and dynamic experiences of non-verbal cues incorporated in their facial features and body movements (Knapp et al., 2013). In group communication, due to the contribution of cooperation and interaction, non-verbal cues have always played an important role to convey information (Mehrabian, 2017). Accordingly, interacting individuals unintentionally synchronize their non-verbal behavior along many levels of social interaction (Latif et al., 2014), leading to feeling of connectedness and cooperation (Marsh et al., 2009), which in turn may bring significant and positive impacts on group creativity (Chen et al., 2008). Thus, dynamic non-verbal cues arguably represent a source of substantial information on the quality of social interactions and therefore group creativity. Specifically, head movement synchrony (HMS) is a common phenomenon in various human communications, and have been indicated to be a marker of successful communication (Ramseyer and Tschacher, 2014). Regarding the music impact on non-verbal cues, noting the enhancing effect some rhythms can have on group synchronization (Brown, 2000), and therefore cooperation (Wiltermuth and Heath, 2009), previous study by our group has also showed the enhancing effect of music (of all combinations and kinds) compared to no music on HMS (Hosseini et al., 2019). The correlation between HMS and group creativity and possible response of HMS to idea generation had not been addressed in our previous study. To address this deficiency, we aimed to first clarify the relationship between HMS response and idea generation.

Understanding group creativity requires recognizing that both the capacities to contribute divergent ideas and to integrate those ideas are wellspring of group creativity. Recent studies on creative outcomes have noted the importance of divergent input for group creativity, as divergent resources stimulate variety in output, as exposure to a greater diversity of ideas enhances the likelihood that participants will produce innovative outcomes (Harvey, 2014). Contrastingly, idea integration during creative process is also important (Xue et al., 2018), as groups that can integrate divergent input have the potential of engendering extraordinary creativity (Eisenbeiss et al., 2008; Harvey, 2014). Consequently, as the second aim we explored potential contribution of HMS response to the quality of the successive idea generation, especially on the divergent/convergent aspect. In a similar fashion with a recent study that examined how dynamic non-verbal movement may emergently affect quality of speech (Paxton and Dale, 2017), the present study suggests that dynamic HMS, which emerges through exchanges of non-verbal cues in interaction, may likely modulate the quality of shared ideas in a bottom-up manner. Here, we also wanted to introduce the BGM capacity to systemize divergent idea sharing. Elaborating the dynamic aspect of idea generation with the idea quality importance aspect, as the second part of our hypothesis, we assumed HMS would help to induce divergent ideas, while upbeat genre, relating to extraversion, along with positive valence, associated with cooperation, and results in successive divergent idea sharing.

For these two purposes, we augmented the Alternative Uses Task (AUT) for this study as the creativity task. The AUT is originally a measure of individual divergent creativity levels (Guilford, 1967). This study used an adapted version of the AUT, that was expanded to investigate group creativity. The adapted AUT involves communication between participants and requesting participants to discuss their ideas so that each group would reach a common idea via a discussion decision-making approach. We recorded the head movement of participants during discussion for further assessments of HMS along with the records of participants’ answers during the AUT discussion, to acquire raw data on idea generation; as the measure timing of idea generation. The timing of idea generation is important as it allows us to investigate if any possible mutual coordination between HMS and idea generation was present. This data also would aid in examining the potential determining effect of the HMS on the quality of the ideas as well as also us to observe the effect of music types.

## Materials and Methods

### Ethics Statement

The Human Subjects Research Ethics Review Committee of the Tokyo Institute of Technology approved this study procedure. All participants were briefed on the experimental procedure and provided written informed consent prior to participation in the experiment. Participants were paid 3000 Yen as a reward for their time and effort after the experiment.

### Participants

Forty international students were recruited from the Tokyo Institute of Technology (23 males, 17 females; age = 26 ± 3.83) via flyers and online surveys to participate in this experiment. They were from various nationalities. All participants were right-handed with normal or corrected-to-normal vision. Participants answered two online preparatory surveys and were paired in groups of two or dyads (6 female–female; 10 female–male; 4 male–male) based on the results of those surveys. Information regarding the online preparatory surveys is provided in the next section.

### BGM Music Pieces

A previous study have referred to the association between unconscious aspects of personality and music preference (Cattell and Saunders, 1954). However, given that such studies were based on a limited selection of music genres, Rentfrow and Gosling (2003) proposed several major dimensions of music – reflective and complex, intense and rebellious, upbeat and conventional, and energetic and rhythmic – to formulate a systematic account of how music may impact individual behavior. Thereafter, this account organized several well-known music genres along each dimension and compared the correlations between music preference in each dimension, Big Five personality trait scores, and cognitive ability. Their results indicated that the reflective and complex dimension was positively related to openness and self-perceived intelligence, while negatively related to social ability and self-perceived rejection of old fashion ideals. In contrast, the upbeat and conventional dimension was positively associated with extraversion, conscientiousness, and self-perceived physical attractiveness. Furthermore, in this study, the classical genre was related to the reflective dimension, while pop, country, and sound track music genres were related to the upbeat dimension (Rentfrow and Gosling, 2003). In the present study, we fixed our music genre dimension of interest as reflective and complex (hereafter labeled as reflective) versus upbeat and conventional (labeled as upbeat), and selected classical tracks to represent reflective dimension while music tracks in the pop, country and sound track genres to represent upbeat dimension.

On the other hand, positive valence in musical pieces has been reported to correlate with divergent creativity while negative valence was found to relatively hinder creativity levels in previous studies (Thompson et al., 2001). In the present study to determine the valence of musical pieces we referred to the general key of pieces and assigned pieces with major keys as having positive valence while labeling pieces with minor keys as having negative valence.

Combining the genre and valence dimensions, we derived four music categories – reflective negative valence (RN), reflective positive valence (RP), upbeat negative valence (UN), and upbeat positive valence (UP), and compared their effects on group creativity.

### Dyadic Group Formation

In the preparation phase, participants answered two online preparatory surveys. The contents of the first survey concerned participants’ available time to participate in the experiment and their contact details. The second survey was concerned with their subjective evaluation of 100 music pieces, consisting of 25 pieces each from each musical category (RN, RP, UN, and UP), presented in a randomized order. As there is a rhythmic effect of music on body movement, the selected music pieces were controlled for tempo (Kellaris and Kent, 1991) to ensure that the pieces were within the range of [95–105] BMP (beats per minute) on average. A recent discussion over the effect of music familiarity and likability on modulating brain region activity motivated us to control for the familiarity and likability attributes in the presented music stimulus (Pereira et al., 2011). Thus, this study only used tracks that unfamiliar but likable for the both members in each dyad. Participants were asked to rate their perceived mood regarding musical pieces to ensure our music valence selection related to engendering or inducing a mood in the participants. To do so participants were asked to listen to the first fifteen seconds of one hundred instrumental music pieces and rate them in terms of likability on a five-point scale (1 “really disliked” to 5 “really liked”), familiarity on a five-point scale (from 1 “unfamiliar” to 5 “familiar”), and perceived mood individually on a five-point scale (from 1 “felt sad” to 5 “felt happy”). Participants who were available on the same experimental time slot, and who rated at least one matching track from each of the reflective and upbeat genres as low on familiarity (unfamiliar) and likable, and very low on mood (representing sad mood) for negative valence pieces and very high on mood (happy mood represented) for positive valence ones, were placed in dyads and called to participate in the experiment. Furthermore, we controlled the intensity of selected pieces to control for participants perceived arousal (Ilie and Thompson, 2011).

### Experimental Procedures

**FIGURE 1:**
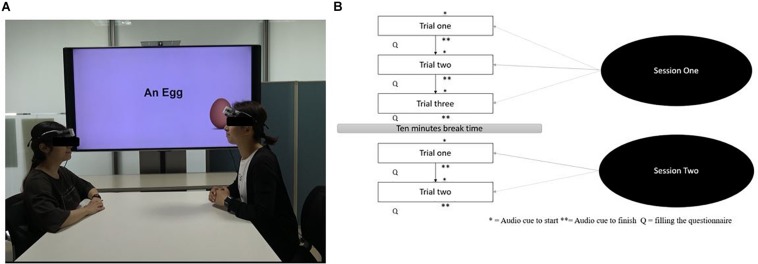
A snapshot of experimental setting **(A)** and the overview of experimental procedures **(B)**.

Participants sat face-to-face with a table in between them and a television screen on their side (experiment setting is depicted in Figure 1). The experimental procedure consisted of two sessions. The first session included three trials and was followed by a ten-minute break and the second session with two trials. Each trial began with an auditory (beep) cue, lasted for a duration of 6 min while the participants completed a group AUT problem, and ended with another auditory cue. AUT is typically a measure of individual creativity. To adapt the AUT for the group creativity measurement, participants were asked to share and discuss the feasibility of their ideas over the alternative usage of familiar objects between each other. On each starter auditory cue, participants were asked to look at the TV screen where name of the AUT stimuli or objects (“1 m of cotton rope,” “An egg,” “A plank of wood,” “A tennis ball,” and “A pair of socks”) were presented. Participants had to try discuss as many ideas as possible and to try be as original as possible when imagining and discussing alternative uses for the objects presented. In each trial, a musical piece (from one of the four music categories selected in the preparation phase) or no music was played as BGM. The order of the objects and music conditions presented in the group AUT task were independently randomized over the dyads. Participants then rated their emotional state (within 50 s) in terms of pleasantness after each trial (the questionnaire results were irrelevant to this particular study).

The experimenter conducted a practice trial with the upbeat positive condition as BGM before the start of the first session to ensure the participants understood the contents of the experiment and that measurement devices were working well. It should be noted that the track used for practice trial was a pre-defined track and differed from the tracks used during main experiment. After the practice trial participants rested for 5 min to reduce any potential effects from this trial on their mood. The task procedure is depicted in Figure 1. To evaluate creative task performance, the experimenter recorded the utterances made during the group AUT using a video camera (FDR-AX40; SONY).

### Assessment of Indices of Creativity Task Performance

We addressed the indices of creativity in three categories of fluency, originality, and index of the convergent (IOC) (Larey and Paulus, 1999). Fluency was the total number of ideas that dyads mentioned during each trial. Idea originality was the function of occurrence of an idea for each object over all groups, where those that occurred in fewer groups indicated higher originality (Dippo and Kudrowitz, 2013).

Firstly, let us denote the frequency count of idea *ID* for an object *OB* over all participant groups as *n*(*OB*,*ID*). Then, the raw originality score of *ID* for *OB* is defined by

O⁢r⁢i⁢g⁢i⁢n⁢a⁢l⁢i⁢t⁢y⁢R⁢a⁢w⁢(O⁢B,I⁢D)=m⁢a⁢x⁢(n⁢(O⁢B,⋅))-n⁢(O⁢B,I⁢D)

Here, *function*(*n*(*OB*,⋅)) indicates the function [*maximum*, *mean*, *andSD* (*asshownbelow*)] of frequency of idea(s) over all groups. Note that the distribution of {*OriginalityRaw* (*OB, ID*) *| ID* in the set of all ideas for object *OB* generated by the all groups} can be very different between objects. For example, an *OB* might have lower max (*n*(*OB*, ⋅)) than other objects, meaning less common ideas for that particular *OB*. Then, the range of *OriginalityRaw (OB, ID)* would be narrower. This difference in distribution is problematic, as though the objects were assigned to different BGM conditions over participant groups, we want to probe into the difference in idea originality caused by different BGMs while controlling (and thus excluding) the influence of object assignment. Therefore, we calculate the *standardized originality score* of each idea as

$$O\,r\,i\,g\,i\,n\,a\,l\,i\,t\,y\,Z\,\left(O\,B,I\,D\right)=\frac{O\,r\,i\,g\,i\,n\,a\,l\,i\,t\,y\,R\,a\,w\,\left(O\,B,I\,D\right)-m\,e\,a\,n\,\left(O\,r\,i\,g\,i\,n\,a\,l\,i\,t\,y\,R\,a\,w\,\left(O\,B,\cdot \right)\right)}{S\,D\,\left(O\,r\,i\,g\,i\,n\,a\,l\,i\,t\,y\,R\,a\,w\,\left(O\,B,\cdot \right)\right)}$$

Then, the *mean originality score* of the ideas generated in the trial by a group *GR* for an object *OB* is given by

O⁢r⁢i⁢g⁢i⁢n⁢a⁢l⁢i⁢t⁢y⁢(G⁢R,O⁢B)=m⁢e⁢a⁢n⁢_⁢G⁢R⁢(O⁢r⁢i⁢g⁢i⁢n⁢a⁢l⁢i⁢t⁢y⁢Z⁢(O⁢B,I⁢D⁢_⁢G⁢R))

Here, the mean is calculated only over the ideas generated by the group *GR*.

Index of the convergent is the index of cooperation and idea integration. IOC for a trial is defined as the total number of times a generated idea by a participant remained in the same category as a previously mentioned idea by the other participant in the dyad over the total number of ideas mentioned during that trial.

To decide on the categories, we referred to the general treatment of the objects – e.g., a plank of wood, may produce ideas like bracelets and earrings which were then placed into a category called “accessories.”

### Data Analyses

#### Head Movement Synchronization (HMS) in Communication

To assess the data of head movement and detect head movement synchronization (HMS), a small wireless accelerometer (TSND121; ATR Promotions) with a sampling rate of 10 Hz was attached to a functional Near-Infrared Spectroscopy device (HOT-1000; Hitachi High-Technologies; the results of brain signals were not a matter of interest in this particular study) in the middle of a participant’s forehead. The head movement time series was analyzed using the same method used in prior studies (Thepsoonthorn et al., 2016; Yokozuka et al., 2018). After extracting the raw head movement data from each participant and applied a short-time frequency analysis (window width = 1280 ms and window Increment = 100 ms) (Fraisse, 1982) to acceleration norms, we assessed the amplitude of head movement within a frequency band of 1.0–5.0 Hz. By applying Spearman’s rank correlation, we calculated head motion correlations with time lags within the range of −500 to 500 ms. We used the head movement correlations in two formats. First, we averaged the value of head movement correlations in all frequency bands and time lags as the HMS during that trial. Second, for each second of the duration of each trial, we summed up all HMS in all frequency bands and if the result indicated the occurrence of HMS (i.e., a summation greater than zero), we coded that second as 1; otherwise, the HMS in that second was coded as 0. By doing so, a sequence of 360 s (6 min) of HMS responses was formed for each trial and we called the acquired vector *HMS*_*t*_.

#### Temporal Coordination of Idea Generation and HMS

We hypothesized that HMS takes place more frequently in response to idea generation, reflecting the coordinated bodily expressions (e.g., agreement, evaluation, and co-laughter) to the ideas. If this is the case, we would observe higher chance of HMS within a short time window just after each idea generation than in other moments. This temporal coordination between idea generation and the responding HMS was assessed in the following steps. Step 1 involves the idea generation sequence. Step 2 involves the execution of a surrogate idea generation sequence. Step 3 involves the time lag-based co-occurrence of idea generation and HMS response. Finally, step 4 involves surrogate-based *z*-score of this co-occurrence. We further used *z*-score based analysis to test if the co-occurrence of idea generation and HMS falls out of the null distribution based on the surrogate data, and identified the time lag that provided the most robust co-occurrence detection in terms of the highest departure from the null distribution. Here, selection of this time window setting is important as the selection of an optimal time window setting, through surrogate-based *z*-scores, enables us to identify the most robust temporal coordination. To be more descriptive, the co-occurrence captured with other time windows might be more contaminated by chance or other factors such as the preceding ideas. With the choice of the current analysis, we can claim that the detected idea-to-HMS co-occurrences are least affected with such contaminating factors. We detail each step in the following.

In Step 1, the time series of idea generation *I*_*t*_ is related to the idea generation times during the discussion between dyads in each of the trials. Based on the recorded voices of the participants during the experiment, for each second during 6 min of one trial, if any of the participants mentioned an idea at *t*_*i*_, the idea is noted with *t*_*i*_. Based on this information, the idea generation binary sequence was constructed as such below:

$$I_{t}=\left\{ \begin{array}{c} 1\;t=t_{i}\\ 0\;o\,t\,h\,e\,r\,w\,i\,s\,e \end{array}\right.\;$$

In Step 2, we first captured the time gap between two idea generation times. Where the interval denotes the sequence of this time. With this in mind, we consider that *N* is the total number of ideas, *t*_*i*_ represents the output time of one idea, and *t*_*i+1*_ represents the output time of the following idea. Thus, the interval sequence is shown in the following equation:

$$Intervals=\left\{t_{i+1}-t_{i};i=0,1,\ldots ,N\right\}$$

**FIGURE 2:**
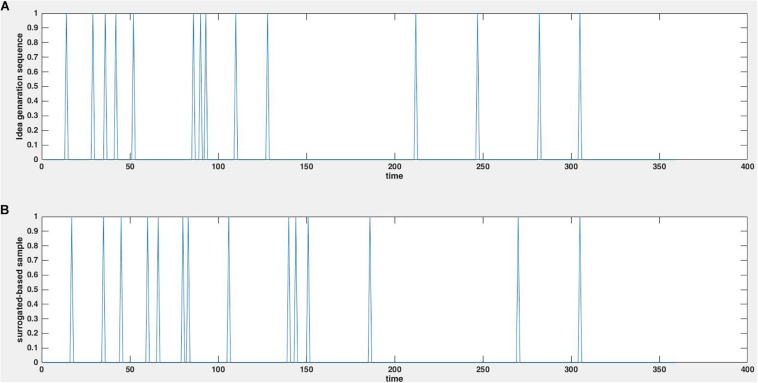
Example of an original idea generation sequence **(A)** and its surrogate **(B)**, which was generated by randomizing the order of idea-to-idea intervals.

Here we used surrogate-based data, in order to characterize group creativity. Surrogating the signals helps in the robust characterization of the system’s behavior and function of any dynamical units. Consequently, the use of surrogate data testing enables robust statistical evaluations to ensure that the observed results are not by chance but are true characteristics of the dynamics. To make the surrogate data, the order of the interval sequence was randomly permutated 1000 times, while maintaining the distribution of the intervals between ideas generated the same as the original distribution. A 1000 surrogate idea generation time series thus obtained is denoted by *SI*_*t*_. Figure 2 depicts a sample of the idea generation sequence and a sample of its surrogate sequence.

In Step 3, set the idea generation sequence as a reference, we captured the HMS within *w* seconds of the generation of a particular idea. For this purpose, we assessed ten time-window settings of HMS sequences within [0, *w*] (*w* = 1–10) seconds from idea generation. Taking *HMS*_*t*_ as the original sequence of the head movement time series for each trial, the *w* seconds time window adopted sequence of head movement synchrony *HMSW*_*t*_ was formulated as:

$$H\,M\,S\,W_{t}=\left\{ \begin{array}{c} 1\;H\,M\,S_{t},H\,M\,S_{t+1},\ldots ,H\,M\,S_{t+w}=1\\ 0\;o\,t\,h\,e\,r\,w\,i\,s\,e \end{array}\right.$$

**FIGURE 3:**
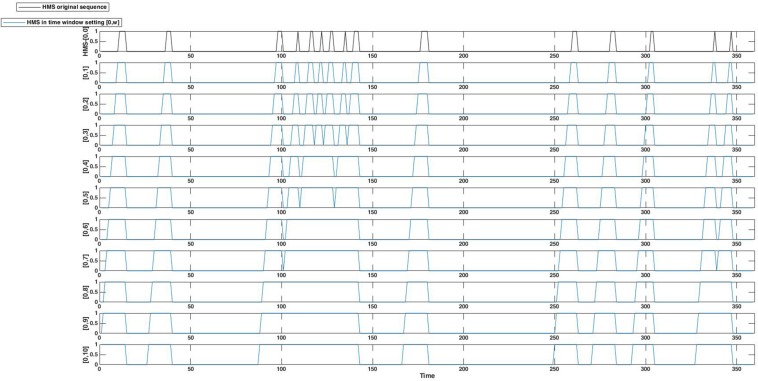
An example of original HMS **(top)** and its window-adopted versions, with 10 time window settings [0, *w*] (*w* = 1–10).

Figure 3 shows the *HMSW*_*t*_ within ten-time window settings.

Furthermore, to assess the co-occurrence of idea generation and HMS response, we calculated the dot product of *I*_*t*_ and *HMSW*_*t*_ as *co*_*oc*_*w*_, along with the dot product of *SI*_*t*_ and *HMSW*_*t*_ as *Sco*_*oc*_*w*_. These can be summarized as:

$$c\,o\,\_\,o\,c_{w}=\left(I_{t},H\,M\,S\,W_{t}\right),$$

$$S\,c\,o\,\_\,o\,c_{w}=\left(S\,I_{t},H\,M\,S\,W_{t}\right)$$

Based on these steps we calculated surrogate-based *z*-scores of the summary co-occurrence of idea generation and HMS below:

$$z-s\,c\,o\,r\,e=\frac{c\,o\,\_\,o\,c_{w}-m\,e\,a\,n\,\left(S\,c\,o\,\_\,o\,c_{w}\right)}{S\,D\,\left(S\,c\,o\,\_\,o\,c_{w}\right)}$$

In the Step 4, we averaged the surrogate-based *z*-scores of the co-occurrence over the five BGM conditions (including no music) for each dyad as the following:

$$m\,e\,a\,n-z-s\,c\,o\,r\,e=\frac{\sum_{i=1}^{5}{\left(z-s\,c\,o\,r\,e\right)}_{i}}{5}$$

Finally, to test whether idea generation was accompanied by lagged HMS beyond chance, we performed a one-sample *t*-test on the mean-*z*-scored co-occurrence values of the 20 dyads for each window setting *w*. Also, comparing the *t*-values we identified the window setting *w* with the highest *t*-value as the optimal time window that captures the co-occurrence of idea generation and HMS response the most robustly. From this point onward, we only used the time window setting with the highest *t*-value to capture HMS from idea generation.

#### Relationship Between HMS and Convergent/Divergent Nature of Ideas

Recent theoretical and practical advances regarding sharing different kinds of ideas to facilitate group creativity motivated us to consider relationship between HMS and the nature of ideas (Harvey, 2014).

Harvey (2014) tried to identify factors that facilitate both divergent and convergent creativity in groups by modulating groups and their individual tasks. Here, we tried to use music as a means to improve group creativity outcomes by fostering divergent idea sharing and the integration of diverse ideas.

For this purpose, two idea attribution vectors, *C*_*j*_ and *O*_*j*_, were introduced, where *C*_*j *_ represents the convergence of each idea denoted by *I*_*j*_, and *O*_*j*_ represents the originality of idea *I*_*j*_. As defined above, originality *O*_*j*_ is negatively proportional to the count of the ideas being generated for each stimuli object by different groups. Here the constant factor of *O*_*j*_ does not change the later result due to the normalization applied below. Given that the idea occurrence count was *n*, groups was *ID*, and object was *OB*, and the total ideas generated during each trial was *N*, *C*_*j*_ and *O*_*j*_ were formulated as below:

$$C_{j}=\left\{ \begin{array}{cc} {{}_{0}}^{1} & I\,\left(t\right)\,i\,s\,C\,o\,n\,v\,e\,r\,g\,e\,n\,t\,o\,t\,h\,e\,r\,w\,i\,s\,e\;f\,o\,r\,j=1,2,\ldots ,N \end{array}\right.$$

$$O_{j}=-n\;f\,o\,r\,j=1,2,\ldots ,N$$

**FIGURE 4:**
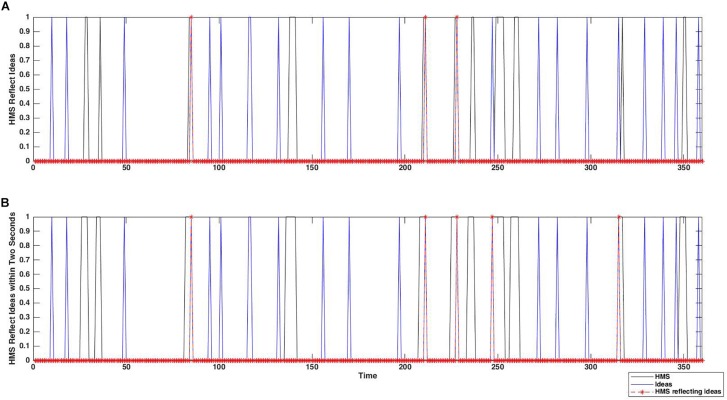
An example of co-occurrence relationship between idea and HMS sequences, with the original HMS series **(A)** and the window-adopted HMS series with time window [0,2] **(B)**.

Furthermore, we introduced a binary idea-HMS co-occurrence series *Vco*_*oc*_*wj*_ as the logical product of the elements of *I*_*t*_ and *HMSW*_*t*_ (i.e., for an idea *j* with *I*_*ti*_ = 1, if *HMSW _ti_* = 1 then *Vco*_*oc*_*wj*_ = 1, otherwise *Vco*_*oc*_*wj*_ = 0). Figure 4 shows the *Vco*_*oc*_*wj*_ for the time window [0,2] in contrast to the idea generation and original HMS. In the next step, we calculated the zero centered normalized vectors of *Vco*_*oc*_*wj*_,*C*_*j*_, and *O*_*j*_. For a vector *V*_*j*_, the normalized vector*norm*_*V*_*j*_ was given by:

$$n\,o\,r\,m\,\_\,V_{j}=\frac{V_{j}-m\,e\,a\,n\,\left(V\right)}{S\,D\,\left(V\right)}$$

The aim of this step was to ensure that the times where participants generate a convergent/original idea and HMS reflect this idea generation accurately are treated as times when participants formulate divergent/non-original ideas and there is no HMS reflected. This normalization was conducted to ensure equally balanced contribution from existence and non-existence or high and low values in the idea-HMS co-occurrence and the attributions of the ideas in calculating their temporal relationship measures (defined as below), irrespective of the possible imbalance in the idea-HMS co-occurrence, convergence, and originality within each trial. Furthermore, to observe whether the co-occurrence of HMS depend on the quality of the generated ideas and to test if such a dependence is out of chance level, we first permuted the orders of *norm*_*C*_*j*_ and *norm*_*O*_*j*_ vectors resampling it 1000 times to observe whether the order mattered, and called these permutation-based normalized vectors as *Pnorm*_*C*_*j*_ and *Pnorm*_*O*_*j*_, respectively. In the next step we calculated the *C*_*co*_*oc*_*w*_ as a dot product of idea-HMS co-occurrence series and idea-convergence series, and *O*_*co*_*oc*_*w*_ as a dot product of idea-HMS co-occurrence series and idea-originality series:

$$C\,\_\,c\,o\,\_\,o\,c_{w}=\left(n\,o\,r\,m\,\_\,V\,c\,o\,\_\,o\,c_{w\,j},n\,o\,r\,m\,\_\,C_{j}\right)$$

$$O\,\_\,c\,o\,\_\,o\,c_{w}=\left(n\,o\,r\,m\,\_\,V\,c\,o\,\_\,o\,c_{w\,j},n\,o\,r\,m\,\_\,O_{j}\right)$$

**FIGURE 5:**
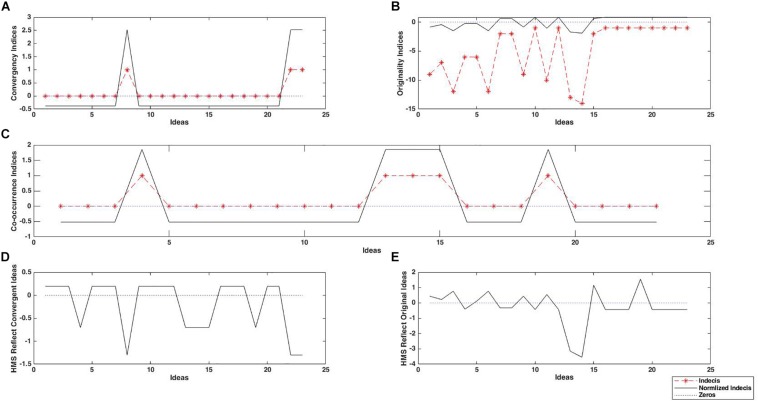
Examples of convergence attribution vector **(A)**, originality attribution vector **(B)**, and the idea-HMS co-occurrence vector **(C)** from a single trial. In each panel, black solid lines represent raw scores, and red dashed lines represent normalized scores. By multiplying the normalized idea-HMS co-occurrence vector with the attribution vectors, relationship between the occurrence of HMS and ideas’ convergence **(D)**, originality **(E)** are evaluated, respectively. By summing up these values over ideas, we obtained summary measures *C*_*co*_*oc*_*w*_ and *O*_*co*_*oc*_*w*_, which represent how much HMS occurrences depend on the convergence and originality of the ideas, respectively.

These variables represent how much HMS occurrences depend on the convergence and originality of the ideas, respectively. Figure 5 depicts these steps. We repeated the same calculation of the dot product between the *norm*_*Vco*_*oc*_*wj*_ and all the 1000 permuted normalized vectors *Pnorm*_*C*_*j*_ and *Pnorm*_*O*_*j*_, denoting them as *PC*_*co*_*oc*_*w*_ and *PO*_*co*_*oc*_*w*_, respectively. In the following step we assessed permutation-based *z*-scores using the following equations:

$$C\,o\,n\,v\,e\,r\,g\,e\,n\,t-z-s\,c\,o\,r\,e=\frac{C\,\_\,c\,o\,\_\,o\,c_{w}-m\,e\,a\,n\,\left(P\,C\,\_\,c\,o\,\_\,o\,c_{w}\right)}{S\,D\,\left(P\,C\,\_\,c\,o\,\_\,o\,c_{w}\right)}$$

$$O\,r\,i\,g\,i\,n\,a\,l-z-s\,c\,o\,r\,e=\frac{O\,\_\,c\,o\,\_\,o\,c_{w}-m\,e\,a\,n\,\left(P\,O\,\_\,c\,o\,\_\,o\,c_{w}\right)}{S\,D\,\left(P\,O\,\_\,c\,o\,\_\,o\,c_{w}\right)}$$

These *z*-scores express in each trial how much occurrences of the HMS as response to ideas have been affected by the convergence and originality of the ideas, respectively.

We also wanted to observe if the HMS response to idea generation would affect the nature of the successive ideas generated. Therefore, in a parallel session, in the two last steps, we shifted the idea-HMS co-occurrence vector *norm*_*Vco*_*oc*_*wj*_ by one idea behind and evaluated its relationship with the convergence and originality of the successive ideas. To do so, first we re-calculated *C*_*co*_*oc*_*wj*_ and *C*_*co*_*oc*_*wj*_ using the shifted *norm*_*Vco*_*oc*_*wt*_ vector. Secondly, calculating and using the *PSHC*_*co*_*oc*_*w*_ and *PSHO*_*co*_*oc*_*w*_ for the respective permutation-based convergent and originality vectors, we derived the *z*-scores with the same formula as depicted above but using the results of the shifted vector and named the additional measures as *SH*−*Convergent*−*z*−*score* and *SH*−*Original*−*z*−*score*. The formulas are depicted below:

$$S\,H\,\_\,C\,\_\,c\,o\,\_\,o\,c_{w}=\left(S\,H-n\,o\,r\,m\,\_\,V\,c\,o\,\_\,o\,c_{w\,j},n\,o\,r\,m\,\_\,C_{j}\right)$$

$$S\,H\,\_\,O\,\_\,c\,o\,\_\,o\,c_{w}=\left(S\,H-n\,o\,r\,m\,\_\,V\,c\,o\,\_\,o\,c_{w\,j},n\,o\,r\,m\,\_\,O_{j}\right)$$

S⁢H-C⁢o⁢n⁢v⁢e⁢r⁢g⁢e⁢n⁢t-z-s⁢c⁢o⁢r⁢e=

$$\frac{S\,H\,\_\,C\,\_\,c\,o\,\_\,o\,c_{w}-m\,e\,a\,n\,\left(P\,S\,H\,C\,\_\,c\,o\,\_\,o\,c_{w}\right)}{S\,D\,\left(P\,C\,\_\,c\,o\,\_\,o\,c_{w}\right)}$$

$$S\,H\,\_\,O\,r\,i\,g\,i\,n\,a\,l-z-s\,c\,o\,r\,e=\frac{S\,H\,\_\,O\,\_\,c\,o\,\_\,o\,c_{w}-m\,e\,a\,n\,\left(P\,S\,H\,O\,\_\,c\,o\,\_\,o\,c_{w}\right)}{S\,D\,\left(P\,S\,H\,O\,\_\,c\,o\,\_\,o\,c_{w}\right)}$$

It should be noted that the trials that contained no HMS within the identified optimal time window from any of idea generation (three trials), and the trials with no convergent ideas (17 trials), were excluded from the analysis.

## Results

### Effects of Music Type on Creativity Outcomes

**FIGURE 6:**
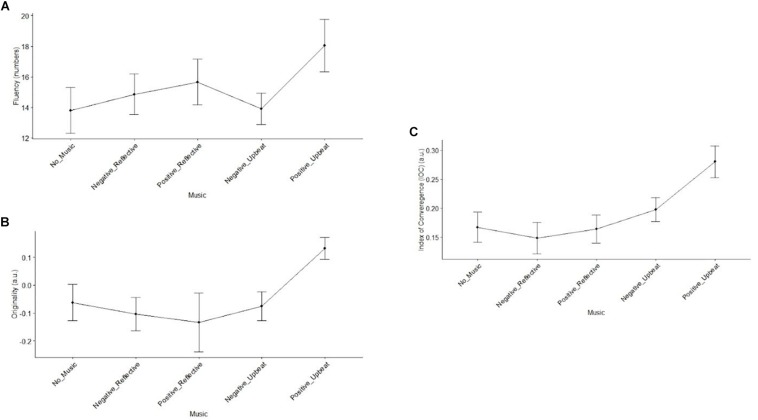
Effects of music types on the group creativity outcomes: fluecny **(A)**, originality **(B)**, and IOC **(C)**. Error bars represent standard error of the mean.

To observe the possible effects of the music conditions on creativity performance, we used R statistical computing software. A one-way repeated measures ANOVA performed on the five music conditions showed significant differences across conditions on fluency (*F*(4,76) = 3.566, *p* = 0.010), originality (*F*(4,76) = 2.93, *p* = 0.025), and IOC (*F*(4,76) = 4.95, *p* = 0.001). Two-way repeated measures ANOVA on fluency, using genre and valence as factors, while excluding the data of the no-music condition indicated significant main effects on valence (*F*(1,19) = 7.81, *p* = 0.012). Yet no significant effect was observed on the interaction term (i.e., valence and genre) and the main effect of genre with respect to fluency. On the other hand, a significant main effect in genre (*F*(1,19) = 10.324, *p* = 0.005) with respect to originality was observed. In addition, there was also a marginally significant interaction between the two factors (*F*(1,19) = 3.30, *p* = 0.085) but no main effect of valence with respect to originality. Contrastingly, a two-way repeated measures ANOVA (using the same aforementioned terms) on the IOC score revealed a significant main effect of genre (*F*(1,19) = 10.49, *p* = 0.004) and valence (*F*(1,19) = 4.82, *p* = 0.041), with upbeat genre tracks associated with higher IOC scores. No significant interaction (i.e., valence and genre) was observed on IOC. Observing these results, separate pairwise *t*-tests were performed between positive-upbeat music and no music control condition, and significant enhancing effects were observed on fluency (*t*(14) = 6.39, *p* = 0.02), originality (*t*(19) = 10.03, *p* = 0.005), and IOC (*t*(19) = 8.40, *p* = 0.009). The creativity performance indices are illustrated in Figure 6.

### Effect of Music Type on HMS

**FIGURE 7:**
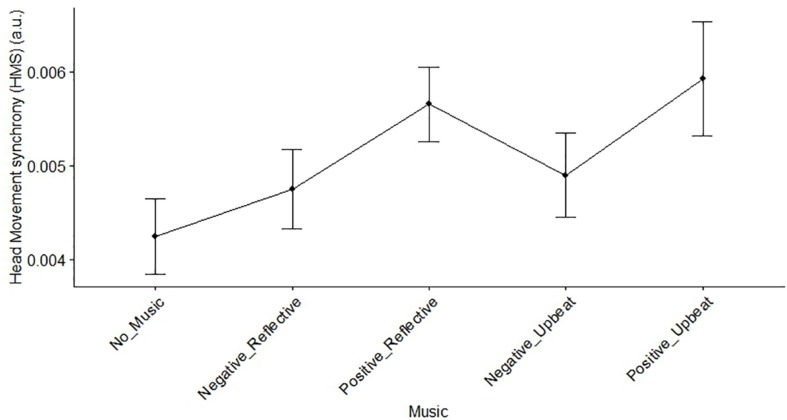
Effect of music types on HMS. Error bars represent standard error of the mean.

To test the effect of music of non-verbal communication we evaluated HMS during each trial of each dyad. To examine the effect of music on HMS, a one-way repeated measures ANOVA was conducted on five conditions. A significant difference was observed across the conditions (*F*(4,76) = 2.58, *p* = 0.043; Figure 7). In addition, a two-way repeated measures ANOVA (factors – genre and valence) was conducted and a significant effect was observed on valence (*F*(1,19) = 4.60, *p* = 0.045), but not on genre or the interaction term with respect to music type.

### Temporal Coordination of Idea Generation and HMS

**FIGURE 8:**
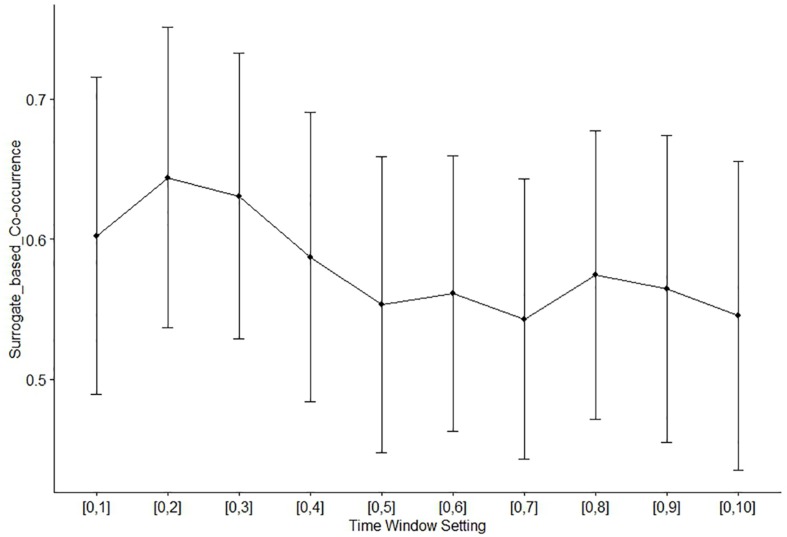
Surrogate-based *z*-scores of the idea-HMS co-occurrence, with 10 time window settings [0, *w*] (*w* = 1–10). Error bars represent standard error of the mean.

In order to test our hypothesis that HMS takes place more frequently in response to idea generation, we identified occurrence of HMS within the time windows of [0, *w*] (*w* = 1 to 10) seconds from idea generation, and evaluated how such co-occurrence (allowing time delay *w* for response) is above chance level, using the permutation-based surrogate data analysis (see section “Materials and Methods” for detail). For each time window, one-sample *t*-test was conducted on the 20 surrogate-based *z*-scores for co-occurrence (obtained one for each dyad, by averaging the *z*-scores over the music conditions). The results revealed the highest level of temporal coordination between idea generation and HMS with the time window setting of [0,2] seconds (*t*(1,19) = 7.00, *p* < 0.0001; Figure 8), indicating that this time window is optimal in capturing HMS as response to idea generation. For the remainder of our results we used this time window setting. Although we expected that HMS would more likely follow idea generation as response, following the reviewer’s suggestion, we also explored the possibility of HMS-preceding temporal coordination with idea generation. Using “negative” time windows HMS within the time windows of [–*w*, 0] (*w* = 1–10) with reference to idea generation as zero, the same analysis showed much lower degree of co-occurrence between HMS and idea generation (Supplementary Figure S1). This result further supports our hypothesis that significant proportion of HMS took place as responses to idea generation.

**FIGURE 9:**
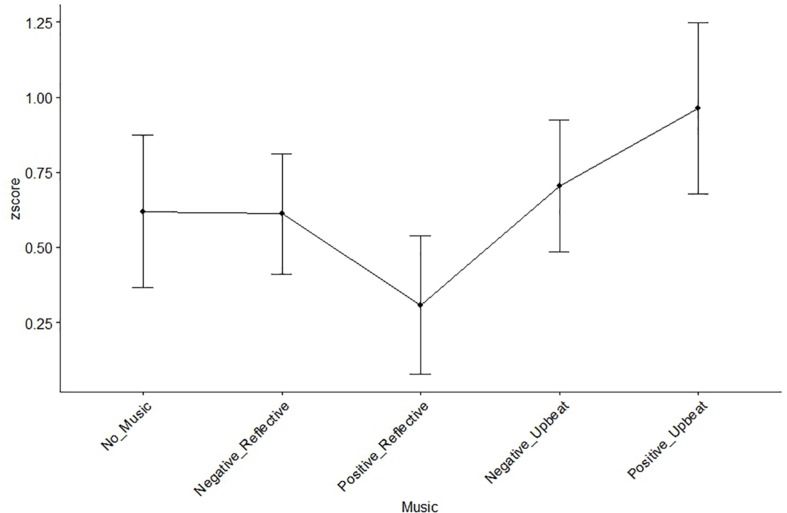
Effect of music on temporal coordination between HMS and idea generation. Error bars represent standard error of the mean.

Furthermore, to check whether the temporal coordination between idea generation and HMS show any difference between the BGM conditions. Excluding data of sessions with no co-occurrence between HMS and idea generation, a one-way repeated measures ANOVA was conducted on the five music conditions and showed no significant difference over conditions (*F*(4,73) = 0.92, *p* = 0.45; Figure 9). Subsequently, a two-way repeated measures ANOVA (genre and valence) was conducted and a significant main effect was observed on genre (*F*(1,18) = 5.73, *p* = 0.028), but not on valence and the interaction term.

### Nature of Previously Generated Ideas and HMS Responses

To examine whether the HMS depended on the quality of previously generated ideas, we averaged the *Convergent*−*z*−*score* and *Original*−*z*−*score* over the five BGM (including the no-music condition). A one-sample *t*-test on the 20-mean permutation-based *z*-scores illustrated no general tendency of HMS as a function of either the convergent (*t*(1,19) = −0.10, *p* = 0.92) or original (*t*(1,19) = −0.22, *p* = 0.82) nature of previously generated ideas. To examine any potential difference with respect to the conditions, a one-way repeated measures ANOVA on the five BGM conditions was conducted. The results showed no significant difference over BGM conditions in respect to convergence (*F*(4,59) = 0.78, *p* = 0.545). Yet, a significant difference was observed on originality (*F*(4,68) = 2.74, *p* = 0.035; Supplementary Figure S2).

### HMS and the Nature of Successive Generated Ideas

**FIGURE 10:**
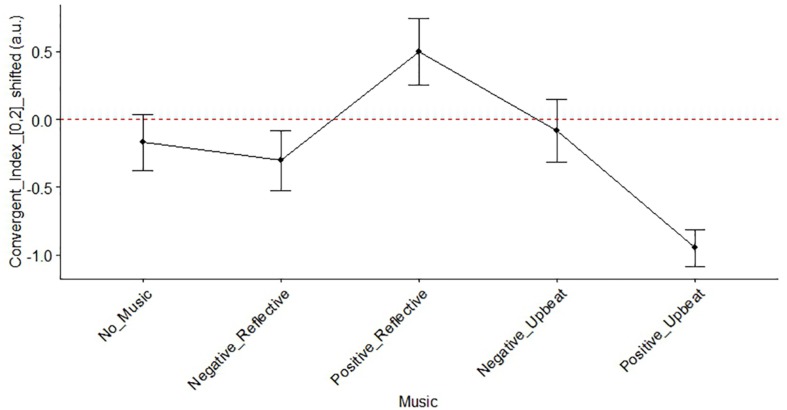
The degree to which a HMS response to idea generation affected the convergence/divergence attribute of the successive ideas generated, for the different music conditions. The horizontal axis shows the score of *SH*−*Convergent*−*z*−*score* in the main text. Error bars represent standard error of the mean. *SH*−*Convergent*−*z*−*score*.

Averaging the *SH*−*Convergent*−*z*−*score* and *SH*−*Original*−*z*−*score* gained from shifted *norm*_*co*_*oc*_*wt*_ over five BGM conditions, we could assess the impact of HMS response on the nature of successive generated ideas. A one-sample *t*-test on the 20-mean shifted permutation-based *z*-scores showed the general tendency of HMS associated with the successive divergent idea generation (*t*(1,19) = −2.44, *p* = 0.024) along with a quasi-significant general tendency of HMS associated with successive original idea generation (*t*(1,19) = 2.02, *p* = 0.058). A one-way repeated measures ANOVA was conducted on music condition and showed significant differences on the convergent (*F*(4,59) = 6.51, *p* = 0.00021; Figure 10) but not the originality attribute (*F*(4,68) = 1.15, *p* = 0.342). In addition, a two-way repeated measures ANOVA (i.e., genre and valence) was conducted on the convergent attribute. The two-way repeated measurement ANOVA revealed significant main effects on genre (*F*(1,12) = 10.05, *p* = 0.008) and a significant interaction between the valence and genre (*F*(1,12) = 10.63, *p* = 0.07). However, no main effect of valence on the convergent attribute was observed (*F*(1,12) = 0.41, *p* = 0.536).

## Discussion

### BGM Effect on Group Creativity

To confirm the previous results regarding effects of BGM on group creativity indices (Hosseini et al., 2019) with a larger sample, we investigated whether listening to a specific type of music as compared to no music control condition might enhance group creativity. The first part of our results indicated a significant main effect of BGM on group creativity in terms of indices of idea fluency, originality and IOC. The main results indicated that the music condition enhanced the fluency of group creativity outcome relative to the control (no music) condition and upbeat genre enhanced the group creativity in terms of originality and IOC. The supplementary results indicate the effect of positive valence in respect to increasing the total frequency of ideas mentioned, while upbeat genre improved the originality of the ideas generated by participant dyads in addition to the IOC as the tendency of the groups to integrate ideas. The effect of positive valence music on fluency could arguably be explained by the constructive effect of positive mood in enhancing ideation component of creativity, such that participants could generate more ideas along with the mood alteration (Harvey, 2014). In terms of originality, no main effect of valence was observed, though contrastingly, a significant main effect was observed for genre. The findings on IOC provided evidence for the benefit of listening to upbeat genres as BGM on idea integration. In conclusion, upbeat positive music have facilitated group creativity outcome, with upbeat genre helping the originality of ideas and positive mood leading to higher scores in the dimension of fluency in ideation.

By comparing a non-verbal communication measure (HMS) of group communication process between the conditions, this study indicated that exposure to BGM enhances synchrony of head motions, which is in accordance with an observation made in a real-world situation (Ellamil et al., 2016).

The overall results of both creativity outcome as well as HMS, suggest that positive valence has an effect on cooperation and integrated communication within dyads, whilst upbeat genre might facilitate the idea integration, leading into the original idea built up. Furthermore, the significant effect of positive valence tracks on HMS enhancement leads us to discuss over the enhancing effect of positive mood on connection between dyads. With such kind of a consideration, we further investigated the relationship between HMS and idea generation during group creativity dynamics, and how the relationship was affected by the types of BGM.

### Generated Ideas and HMS Responses

Examining the HMS response in relation to previously generated ideas, the contribution of non-verbal social behavior during conversation, and the synthesis of interpersonal movement synchrony responses in conversation, has produced great insights into conversation in previous studies. To be specific, movement synchrony is recognized as a contributing factor to rapport (Lakens and Stel, 2011), which can lead to cooperation, during social exchanges. Our results on the temporal coordination provide support for the presence of a relationship between idea generation and HMS response, which was more prominent with the upbeat genre. This finding could be possibly explained by the desire to connect during social interaction (Horowitz et al., 2006), where synchronized head movements may be an embodied expression of agreement with the generated ideas. Also, the prominent association of genre gained in the results might suggest upbeat genres encouraged dyads to participate in the task and behave task related at a higher level. On the other hand, when all BGM conditions were combined, we did not find significant general relationship between HMS and the quality of the previously generated ideas. Consequently, it might be valid to suggest that during creativity related experiments, participants would likely show some levels of understanding and agreement on the task (Shockley et al., 2003) and engagement in the task, regardless of the quality of the generated ideas.

### HMS and Successive Idea Generation

In this study, as a part of testing the second hypothesis, we investigated how the HMS responses would induce participants to share divergent perspectives and original ideas in the successive idea generation. The findings indicate the significant impact of HMS in encouraging dyads to generate more divergent and original ideas. In other words, the chance of such idea generation would increase after dyads expressed some level of agreement on the previously generated ideas. In general, divergent and original ideas are valuable resources during creative group tasks, as has been indicated by the importance of sharing divergent ideas that has been reported in numerous previous articles. Previous research on creativity suggest that group creativity is stimulated by diverse membership (Runco, 1993). Also, previous research on the individual differences in understanding indicate the role of how integrating the diversity of group members enhances decision making (Maznevski, 1994). In sum, it is important to identify what kind of element in the creative communication can promote sharing of more divergent and original ideas. Hence, the relationships between HMS and the nature of generated ideas, specifically the divergence of ideas, are arguably the most important findings produced in this study.

In the other part of our second hypothesis, we were also interested in the potential effect of music type in enhancing this effect further. As for this point, the differential contribution of successive divergent idea generation during upbeat genre BGM (relative to reflective music and a control condition) suggests the role of this genre in facilitating divergent idea sharing. To support this finding, we can refer to the distinct characteristics of the upbeat genre on the embodied extraversion of dyads during group creativity which have facilitated divergent idea sharing. This complementary result provide an interoperation that upbeat positive valence encouraged to share more divergent idea following agreement on the previous idea between group participants. Previously we mentioned the effect of music with a positive valence on enhancing individual creativity and cooperation (Hosseini et al., 2019). The current findings provides mechanistic description of how the positive upbeat BGM enhanced interpersonally coordinated head motions, possibly reflecting increased expression of agreement, and in turn led to generation and sharing of more divergent ideas.

### General Discussion

Altering mood is one of the most studied effects of music in the literature (Gerrards-Hesse et al., 1994; Balch and Lewis, 1996), while the literature on the group creativity suggests there are numerous disadvantages (e.g., social anxiousness and social loafing) to group activities that will likely impact group creativity. In addition to these widespread production blocks, behaviors such as judging and evaluative behavior have been noted as limitations that can impede the creativity of group sessions (Sternberg and Lubart, 1991). Previous studies have demonstrated the effects of nominal groups (Paulus and Nijstad, 2003) or virtual groups (Elias et al., 2011) to address the issue. However, this paper utilized BGM in an attempt improve creativity during group sessions. Here we suggest that music would decrease the stress level during the group creativity task by influencing participants moods (Fried and Berkowitz, 1979; North and Hargreaves, 1999; Ratcliffe et al., 2013). Contrastingly, positive mood results in the increased generation of ideas and cooperation, while the upbeat genre increased motivations to share ideas and incorporate diverse input into the creative group session. As such, this resulted in higher levels of total creative performance in the fluency and originality of the ideas produced.

### Limitations

The primary limitation of our study is that our participant sample was based on a variety of cultures and nationalities. This would induce different body movements in synchronicity (Ekman, 1977) so much that such synchronized body movement might not be always observed during idea sharing. Furthermore, the potential effect of language as a barrier while sharing ideas could be considered a limitation to this study. Furthermore, this study did not control for gender (Pearsall et al., 2008) nor familiarity of groups members and future studies should ensure they account for these two effects as it may bring further insight into how BGM influences group creativity.

## Conclusion

As observed in the findings of this BGM has the capacity to improve group creativity performance – with findings that indicated higher amounts of shared ideas and increased originality relative to no music in the positive-upbeat condition. Upbeat positive music embodied this enhancing behavior the most. We observed that positive valence can enhance HMS and purposed the effect of a positive mood to facilitate cooperation level. We also stated that an upbeat genre might have enhanced the level of idea integration that was encouraged by engendering divergent perspective sharing. Our first claim was due to a higher level of IOC observed during upbeat music and the fact the HMS during idea generation affected the quality of successive ideas produced. For this purpose, we first addressed the temporal coordination of HMS and idea generation. Significant levels of synchronized head movements contributed to the ideas generated during the group creativity task. The upbeat genre music appeared to embody this effect the most, given the results.

In terms of HMS being related to specific kinds of ideas, a general tendency toward agreement on ideas leading to subsequent further sharing of divergent and original ideas was observed. This tendency of sharing divergent ideas was most significant during upbeat music with a positive valence. In the first part of this study, we argued for the possible enhancing effect this music condition could have on group creativity in terms of both fluency and originality. The HMS corresponding responses arguably represented a form of embodied cognition in relation to the sharing divergent resources after agreement on ideas was established during the most upbeat and positive valence tracks. This may illuminate the effect of this BGM type in respect to its impacts of divergent idea generation and integration to induce extraordinary group creativity.

## Data Availability Statement

The datasets generated for this study are available on request to the corresponding author.

## Ethics Statement

The studies involving human participants were reviewed and approved by the Human Subjects Research Ethics Review Committee of the Tokyo Institute of Technology. The patients/participants provided their written informed consent to participate in this study.

## Author Contributions

SH designed and conducted the experiment, analyzed the data, and wrote the manuscript. XD helped with conducting the experiment. YM provided conceptual advice on the experiment. TN designed and supervised the study and experimental design, as well as provided advice on the analytical methods, results, and overall manuscript. All authors discussed the results and commented on the manuscript.

## Conflict of Interest

The authors declare that the research was conducted in the absence of any commercial or financial relationships that could be construed as a potential conflict of interest.
