# Supplementary material for: Head Movement Synchrony and Idea Generation Interference – Investigating Background Music Effects on Group Creativity
Source: Front Psychol. 2019 Nov 15;10:2577. doi: 10.3389/fpsyg.2019.02577 (PMC6873777; doi:10.3389/fpsyg.2019.02577)
Supplement: Supplementary file 1 [file Data_Sheet_1.pdf]

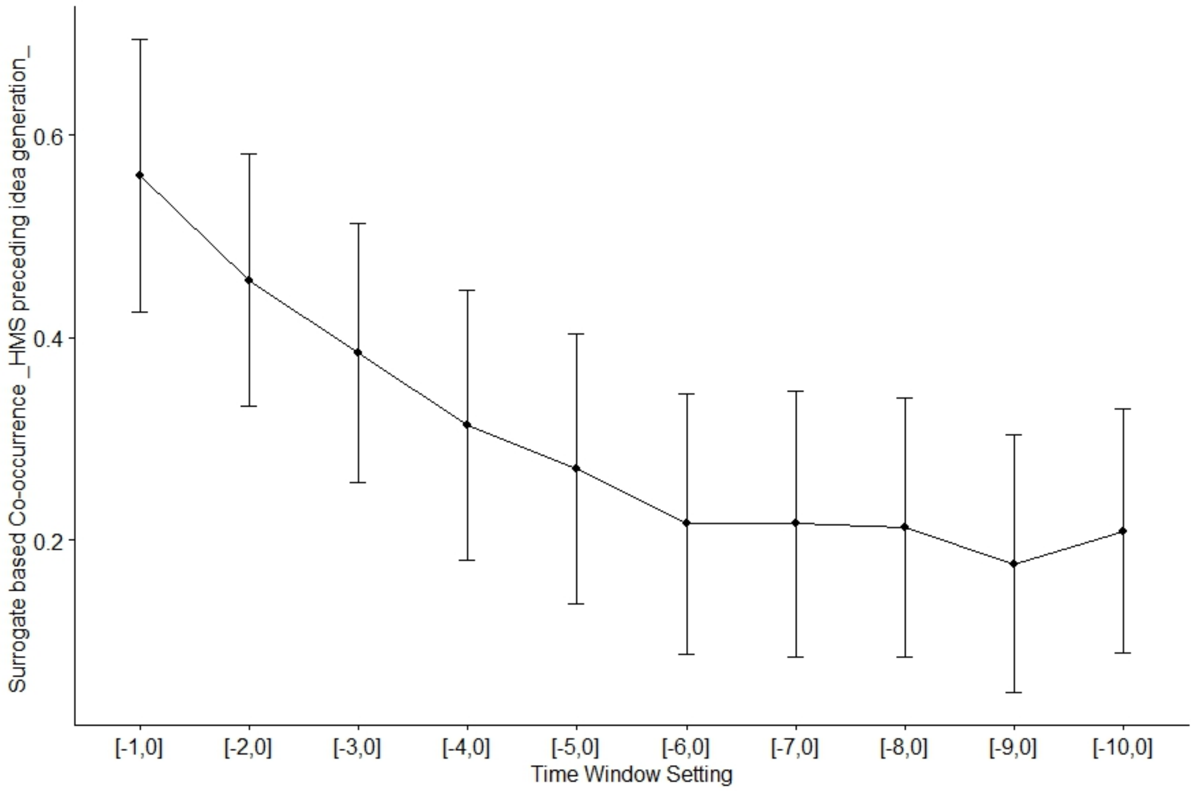

Figure S1. Surrogate-based z-scores of the HMS preceding idea generation co-occurrence, with ten time window settings [-*w*, *0*] (*w* = 1 to 10). Error bars represent standard error of the mean.

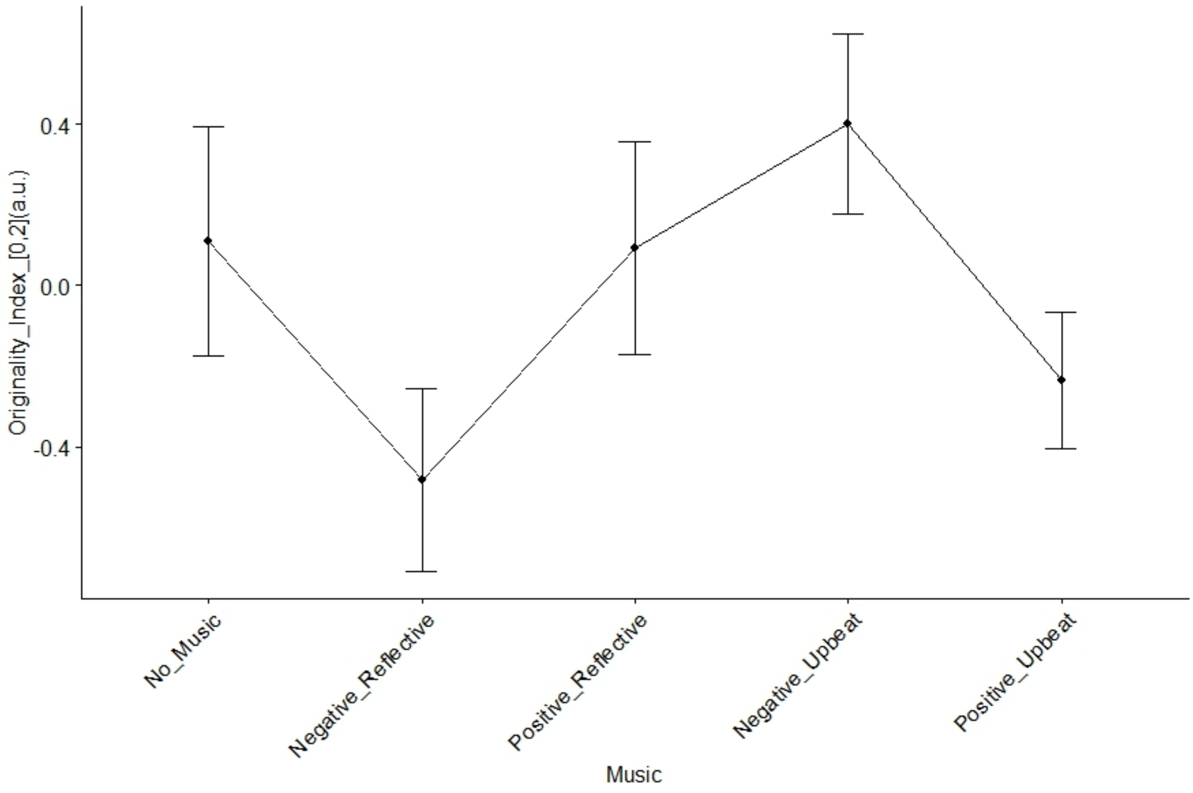

Figure S2. The degree to which a HMS response to idea generation affected the originality attribute of the ideas generated, for different music conditions. The horizontal axis shows the score of $SH\_Original - z - score$ in the main text. Error bars represent standard error of the mean.
